# Calomel in Diseases of Children

**Published:** 1854-03

**Authors:** Samuel Jackson

**Affiliations:** Northumberland


### MEDICAL EXAMINER.

NEW SERIES.—NO. CXI.—MARCH, 1854.

### ORIGINAL COMMUNICATIONS.

*Calomel in Diseases of Children.* Read before the Philadelphia County Medical Society, October 12th, 1853. By SAMUEL JACKSON, M. D., of Northumberland.

Dr. S. Jackson (Northumberland) having been requested to prepare a paper on this subject, which might stimulate to discussion, read the following:—

As a great river has many fountains and confluent streams, so has the subject of which we are called to treat. We have all been exploring these sources, but have all gone some parts of the way in too much haste and with many embarrassments, often enveloped in darkness, sometimes with minds prepossessed by opinions and impeded by tradition or prejudice. Let us then revive our observations and compare our recollections, that our future voyages on these delighting streams of knowledge may be made with greater safety, pleasure and profit.

Your committee desired me to write a few pages that might prove useful in opening the way to this evening's discussions, something that might excite us to a friendly controversy, eliciting each man's private opinions, thus making them common property for the benefit of our patients. Our benevolent profession has many strong alliances to religion; one is, that it may be enlivened and strengthened by the conflict of opinions, all verging to one final and happy consummation.

The task assigned me is difficult, for on *the uses and abuses of calomel in diseases of children,* a writer would not soon come to his end, so manifold are the controversies on this subject, and so extensive is the employment of this powerful agent. In the *first* place, he would have to consider the various supposed modes of its operation; *secondly,* the indications and intentions in giving it; *thirdly,* under what circumstances it may do harm; *fourthly,* the modes of giving it in particular diseases; *fifthly,* the means of counteracting its excessive operation. How small a part of these various points can be attended to on the present occasion, must be seen by you all.

*In diseases of children,* it may be fairly said, that it is principally given as a purge, therefore the various modes of giving it may be considered under the head of particular diseases; but calomel stands pre-eminent as the great alterative, by which term is meant—a medicine that somehow relieves the organism of some embarrassments, and fits it again for the just operation of aliment and stimuli. To this mysterious working of this noble medicine, our attention to-night is particularly called.

Certain it is, that mercury promotes all the secretions, particularly that of bile, that it hastens the absorption of many morbific deposits, that it arrests the deadly progress of inflammation, that it is said to disintegrate the fibrin of the blood so far as to prevent the forming of new and deleterious membranes. These are all prominent desiderata, but whether they can be attained with safety in the diseases of children by the continued use of calomel, is a very serious consideration, not to be neglected in the treatment of each case on hand. For it is too well known to us all, that mercury has sometimes an unexpected and fatal tendency to the maxillæ of children. I do not here allude to the *cancrum oris,* nor yet to the *gangrenous erosion of the cheek,* which I named some years ago *gangrænopsis;* these two distinct diseases are never directly caused by this mineral; but that mercury does attack the gums and alveoli of children, causing gangrene, caries, and sloughing, was known half a century ago, though not perhaps sufficiently regarded at the present time. I have seen three cases of this misfortune in my own practice from the use of calomel. Two children, about seven years old, took this medicine as a purge in remittent fever, and a third child in her fourth year, took it as an anchor to windward in the perils of pneumonia; they all escaped without any external deformity, but each lost two or three molar teeth with their alveoli, after a tedious exfoliation. The fourth case was that of a child about three years old, to whose face I had applied mercurial ointment, in order to prevent the pits of confluent small-pox. All her teeth were loosened, some she picked away in her delirium, the gums mortified, then the cheek, and she soon died of her two-fold disease—an awful sight for the unfortunate physician. She had taken no calomel, hence the disaster must be imputed to the inhalation of vaporized mercury.

Dr. Rush used to caution us against attempting to salivate children under six years old; and, in writing of its producing gangrene in the jaws of children, he says:—"Seven instances of death from that cause, in children between three and eight years old, with circumstances of uncommon distress, have occurred in Philadelphia since the year 1795."* You see then the caution of this great man and heroic practitioner, who has been most unjustly denounced as rash and dauntless in the use of mercury. But to show how timid he was in comparison with some inferior spirits, I will quote a few lines from Professor Beck on *Infant Therapeutics.* "Under various circumstances Dr. Clarke has prescribed mercury in very large quantities, and in a great number of cases, and he never produced salivation, except in three instances, in any child under three years old. Dr. Warren, of Boston, has never known an infant to be salivated, notwithstanding he has given in some cases *large quantities* with this view. Drs. Evanson and Maunsell say, we have never succeeded in salivating a child under three years of age. Dr. Percival repeatedly observed, that very large quantities of ung. cœruleum may be used in infancy and childhood without affecting the gums."

You then see that foolhardy and unjustifiable attempts to salivate children are often made; and you see, too, that never were doctors more fortunate than these, supposing they have told us

* Inqs. and Obs., II., 242.

the whole truth. Mr. President, had I written to you from Northumberland, that I was in the practice of giving mercury to young children in this amount, and with this intention, you would have said, "Oh, he does as he pleases back there, he has no one to watch him, perhaps he knows no better; that backwoods doctor would do well to look into Prof. Beck's little book, p. 45, where he may read thus:—'What shows incontestibly that the action of mercury is actually more energetic on the infant than on the adult, is the fact, that when salivation does take place in the infant, as it sometimes does, its effects are most disastrous. Sloughing of the gums and cheeks, general prostration and death, are by no means uncommon occurrences. On this subject Dr. Blackall justly remarks—a general opinion prevails that the constitutions of young subjects resist mercury. Its entrance into the system they do resist more than we could expect, but they are greatly overcome by salivations; and the possible occurrence of such accidents may well set us on our guard. Dr. Ryan, too, says that ptyalism of infants is often followed by sloughing of the gums and cheeks, and this I have known to occur after the use of it in hydrocephalus.'" I was not wrong then in calling those doctors *fortunate*, using this word in the hope that they concealed nothing—in the hope that if any of these children suffered gangrene of the jaws and face, they would have told us so.

The recollection of such cases must forever deter us from giving calomel to children, for many successive days, as a deobstruent and alterative; hence we suffer great loss in the treatment of many diseases, as pneumonia, obstructions of the liver and spleen, encephalous inflammation, dysentery, and fevers with cerebral oppression. In treating an adult, we detect in good time the mercurial breath, but in children, the gums are swelled and the teeth are loosened, before we have reason to suspect the latent and now irresistible evil. Moreover, the adult jaws can bear more inflammation than those of children, and the patients themselves are more tractable.

Notwithstanding my timidity and the causes thereof, which I have freely expressed above, calomel is a favorite medicine with me. I have given it freely in the diseases of children, and I recollect no unpleasant effects therefrom during the last thirty years, those three cases of maxillary disease having occurred before this period; nor can I recollect having salivated any person the last thirty-four years to such a degree, that he could not have chewed his beef without flinching or pain. Yet in conversation and practice with my brethren, I find that too many of them consider me as rather incautious in the use of calomel. It must be confessed that I have often given it with fear and trembling, nor has my fortunate escape for so many years had any effect in fortifying me against the maxillary panic.

Whoever gives calomel to children every day, ought to be convinced that he has imperative reasons therefor; he must beware of cold air and water;* he ought to examine the breath and gums two or three times every day; and he must warn the mother of what he is doing. Let him also consider the various states of the system that favor or oppose the operation of mercury on the maxillæ. "If we suppose," says Dr. Armstrong, "that the action of medicine is the same in disease as in health, we shall often be greatly mistaken, and particularly in respect to calomel; a few grains of this medicine will usually affect the mouth in health, whereas in fevers attended with a hot and dry skin, we may give full and repeated doses without any such result." 'Tis true that when the system is occupied by any considerable fever, the mercury is not very apt to bring the jaws under its influence, but this preventive fever is often soon reduced, and the body is now open to the action of the accumulated doses of calomel. This would be very apt to happen in pneumonia, the inflammation having been brought low by the appropriate bleeding, starving, and antimony. In dysentery, where small and frequent doses are often greatly needed for some days, one might hope that the intestinal irritation and flux would divert the mercurial action from the jaws, but I have known several instances, though not in my own practice, wherein the maxillæ were seriously injured by calomel in this disease. It is in a state of debility with continual fever, that gangrænopsis is most likely to happen, and to be most intractable. This predisposing state is found in dysentery, in typhus and in remittent fevers, all which may preclude

* In the debate that followed, one gentleman of great authority in medicine, expressed surprise that I should have considered cold as conducing to sore jaws. There is generally some reason for old prejudices, and they oughtnot to be hastily despised.

for some days the salutary operation of bark, quinine, and animal food. In this low state with continual fever, a very slight irritation may dispose the cheek to gangrene; I have seen it evidently brought on, in two cases of this state of weakness and fever, by the pressure of the molar teeth, the patients having laid continually on the same side, with a hand between the cheek and the pillow. Now, there is reason to fear that calomel affecting the jaws, may irritate the cheek, and thus prove the indirect cause of gangrænopsis. This did undoubtedly happen in the child whom I mentioned as affected by vaporised mercury.

Much has been written on the appropriate doses of calomel, and wonderful, indeed, is the discrepancy of the brethren on this subject. The advocates of minute doses argue, that they are as great as the system can profitably digest. In the mode of Dr. Ayre, they would give one-eighth of a grain to an adult, when others would give from one to ten grains. This equality of small to large doses, it must be observed, has no verging towards the homœopathic folly, but it has never been proved, hence it affords a laudable subject for discussion. It is certain, however, that many cases and some idiosyncrasies, abhor large doses of this medicine, and are yet benefitted by such small ones as forty years ago would have been held in contempt. But physicians since the time of Dr. Rush, have so thoroughly learned to think, each one for himself, that every one possessed of a philosophic spirit will fancy, that he sometimes sees indications for small doses, sometimes for large, and in his attention to this subject he will often find cause for self-gratulation. The patient is recovered by nature or by art, and the physician cannot be blamed if he attribute the cure to his own perspicacity. This is a part of our subject that claims the particular attention of every practitioner. The doses of medicines to purge, to puke, or to arrest an intermittent fever, are pretty well ascertained, but the most profitable doses of mercurial alteratives, are not easily settled to general satisfaction.

We are greatly indebted on this subject to physicians of the British army. If any one condemn their practice, he must yet acknowledge, that they have given him much on which he is bound to reflect and experiment. If the European constitution, when melted down by the burning suns and the scalding vapors of the torrid zone, can yet bear scruple doses of calomel, repeated from two to four times in twenty-four hours, and be plainly benefitted thereby, we ought to think at least twice before we speak once against their extraordinary practice. Let us bear in mind, that these are men who deserve well of the whole profession, they have done all they could and are *not* "unprofitable servants;" that while many physicians who have spent long lives in great cities—even some lauded professors—have done nothing for the science, these roving men beset continually by every impediment to study, have furnished us with important facts and principles, have left works that must transmit their names to the love and to the gratitude of distant posterity.

With respect to large doses of calomel, Dr. Cartwright has some peculiar thoughts which I am led to mention, because they are buried in the pages of an obsolete periodical—the Medical Recorder, vol. viii. He supposes that these doses are safe and supremely beneficial, because they act at once upon all the secretions of the body, disposing it to resume the functions of health; whereas small doses often repeated, act merely on the liver. He gave twenty grain doses in dysentery, frequently repeated, and found them, as did the physicians of the British army, not only salutary but absolutely antiemetic and anodyne. He contends that even syphilis is arrested by these, more certainly and quickly than by the small, frequent doses. He says, "experience teaches us, that this decisive method of introducing it, so far from producing mercurial irritation or fever, on which the evils of a mercurial course depend, produces a directly opposite state of the system, by establishing a gentle yet general and permanent secretory action throughout the body."

Dr. Thomas Bond called calomel more than a hundred years ago, "the great revolutionary medicine." Now if a revolution can be wrought, as Cartwright contends, by a few large doses, every one must find himself under a moral obligation to investigate this subject. They are safer with respect to the maxillæ of children than the small and frequent doses, as they appear to work themselves off by all the secretions at once, and thus do all that is expected of mercury.

Amidst the perils and panics of giving calomel, it would be a great relief to find some means of counteracting its possible and much dreaded superaction. Iodine was strongly recommended in Hufeland's Journal, 25 years ago, for the sudden cure of exorbitant salivation; whether it has been further proven, I have not heard. But in No. 21 of the Med. Chir. Review, is a most interesting paper translated from Mon. Melsens, in which this experimenter appears to have proven, that iodide of potassium seizes upon mercury lodged in the living fibre, whether pure or in any possible combination, and carries it suddenly away by the kidneys. But alas, there is no safety even in this, for the poor jaws: the triple compound thus formed, though quickly carried away, has yet time to bring on a profuse salivation, hence it is more dangerous than the metal it was given to expel.

I will now say a few words on the diseases wherein calomel is preeminently beneficial. Among these are considered all the idiopathic fevers, or the cases of them wherein there is a want of bile, or there may be an oppression of the brain present or threatened. The remittent miasmatic fever of children, and the scarlatina, often come on with convulsions, and here a powerful dose of calomel, as soon as the excitement is broken down, must not be omitted. The brain must be thoroughly relieved of its obstruction, lest the convulsions return with the diurnal exacerbation, and there is no medicine that disembarrasses the brain as does calomel in large doses. In scarlatina, even of the mildest grade, I would give two or three calomel purges, for this reason: —these mild cases are as apt as the severe, to be followed by dropsy, suppuration of the ears, paracusis, and glandular swellings continuing a long time, all which may be prevented by low diet, purging, and minute doses of antimony. I am always sorry to find any writer maintaining, as the present fashion is, that "the less we do in such mild cases the better;" I would not run into the opposite extreme, *in contraria*, like Horace's fool, and say the more we do the better, but I do assert from much experience, that there is great good to be done by an active treatment. A case, moreover, which is now mild in the onset, may become very severe. *Respice finem*, says that cautious yet great practitioner, Dr. Rush, should be our motto in every case; let us then hear what Dr. Armstrong says directly to the present purpose. "When in attendance on a family in which scarlet fever existed, I was requested to look at a boy who had just sickened, for whom

I prescribed a laxative and a mild emetic, as the symptoms were then slight; but early next morning I received an urgent message, and to my surprise, found him expiring under coma and convulsions." Other examples of a like insidious nature have come under my observations, and they seemed utterly inexplicable, until I had obtained some morbid examinations, and then the manifest mischief of the visceral congestions, &c."

In a case of open and free reaction, though the fever may run high, the eruption be copious, and the throat be inflamed, there is little danger in trusting the cure to bleeding at the arm, an emetic of ipecacuanha, calomel purges, tartar emetic, ice in the fauces—totally dispensing with a *three week's greasing with the blubber of hogs.* But let me repeat that calomel purges are a great help in preventing the distressing sequelæ of the disease. In the congestive cases it cannot be omitted: and in the malignant form of the disease, whether congestive or not, it often finds a place. There is no medicine that so greatly relieves the congested brain as does calomel. "The power, says Dr. Armstrong, which calomel has of equalizing the circulation, is no where more conspicuously displayed than in diseases of a congestive character." And in another place he says, "though the experience of every succeeding year tends more firmly to establish my faith in the efficacy of large doses of calomel in highly congestive diseases, yet having once made the desired impression they ought not to be repeated, such an extraordinary practice being only requisite or justifiable during the urgency of extraordinary cases." You see then how this great medical hero, shows caution as well as vigor.

Of dysentery, I would hardly undertake to cure a single bad case without calomel. Here you often want more bile—calomel brings it: you need something that serves to arrest inflammation—calomel does this: you want something that powerfully promotes all the secretions—calomel is the great secerner: a remedy is needed that acts most searchingly on the mucous membranes—calomel does it: do you desire some purgative that lies harmless in the bowels till they are ready to be operated upon?—calomel with ipecacuanha and opium, will often answer all your reasonable expectations.

In cholera infantum, we cannot practice without this noble deobstruent. Here the greatest danger lies in the state of the brain and this medicine, as I said before, is the great emulgent of this organ. The most blundering physician, I don't mean homœopaths, can stop a vomiting and purging; but these are not the disease, they are merely symptoms of something worse. Every physician knows that the state of the brain is to be intensely regarded. Only look to the excellent paper of our fellow member, Dr. Hallowell, in Hays' Jour. for July, 1847, and see what encephalic devastation he found in those who died in what he calls the 2d and 3d stages; now this pathological state will be aggravated by stopping the vomiting and purging, unless some salutary evacuations be substituted therefor, and one of these is that universal secretion that calomel affords. I called Dr. Hallowell's paper excellent—I might more justly have used the superlative degree; but with the name he gives this malady, I find cause of complaint. "Gastro-follicular enteritis" is not the disease, but merely a portion of its lesions, and these do not exist if the patient die in the violence of the onset. New names do not help on the cure of diseases.*

Never were calomel and mercurial oint. more used and abused than in the meningitis of children, by which I mean an inflammation in one or all of the encephalous membranes; that disease called hydrocephalus, most beautifully described by Whytt and then by Quin; whether tuberculous, is nothing to our present purpose. Here calomel must be used in very large doses, so as to work itself off by all the emunctories, thus changing the condition of the whole system at once, and making a powerful

* A distinguished member found great fault with this criticism, and tried to justify the name. But the lesions of these follicles, which ought to be called *gastro-entero folliculitis*, so far from being peculiar to chol. infant. are often found in dysentery and enteritis. They are not mortal, the cause of death is found in the brain—congestion, inflammation, and effusion. If the disease has not lasted long enough to produce these, the patient has died of mere prostration. I protest against this naming of idiopathic diseases from their ultimate lesions. A few years ago remittent fever was a *gastro-enteritis*—what is it now? The mild typhus was an *arachnitis*; by the *gens d'armes physiologiques*, it was afterwards called "*the first shade of chronic gastro-enteritis*;" now it is getting the name *enteric fever*. Thus the scarlatina might be called *febris tonsillaris*, and the plague would adopt the euphonious name *febris anthraci-bubonigeneta*.

derivation from the head, according to the views of Cartwright. To give it day after day in small doses, would greatly endanger the maxillæ, particularly as ice or the cold *douche* must often be used to the head. Dr. Rush, in treating of this disease, says,—"my reasons for not giving mercury as a sialogue are, the uncertainty of its operation, its frequent inefficacy when it excites a salivation, and above all, its disposition to produce gangrene in the tender jaws of children:" and Dr. Michael Ryan says—"ptyalism of infants is often followed by sloughing of the gums and cheeks, and this I have known to occur after the use of it in hydrocephalus."

I could say much on the use of calomel in other diseases, were it not high time to hear what my fellow members have to say:—but first, hear the eulogy of Dr. Armstrong, which I heartily adopt. "Only let practitioners put calomel fairly and extensively to the test in febrile diseases, and we shall soon cease to have imaginary and unfounded clamors against its free employment; for here it is a medicine which, like an injured and innocent individual, will have its character restored by an impartial and a strict examination."

